# Charge Trapping Effects on n−MOSFET Current Mirrors Under TID Radiation

**DOI:** 10.3390/mi16091064

**Published:** 2025-09-20

**Authors:** Dorsaf Aguir, Sedki Amor, Laurent A. Francis, Mohsen Machhout

**Affiliations:** 1 Laboratory of Electronics and Microelectronics (LEMM), University of Monastir, Monastir 5019, Tunisia; sedkiamor@gmail.com (S.A.); machhout@yahoo.fr (M.M.); 2 Institute of Information and Communication Technologies, Electronics and Applied Mathematics (ICTEAM), Université Catholique de Louvain (UCL), Place du Levant 3, 1348 Louvain-la-Neuve, Belgium; laurent.francis@uclouvain.be

**Keywords:** n−MOSFET current mirrors, total ionizing dose, electrical stress, threshold voltage, current gain, input−output impedances

## Abstract

This study aims to evaluate the effects of total ionizing dose (TID) radiation on the performance of n−MOSFET current mirrors. We propose an ovel experimental approach to analyze the interaction between charge trapping in the MOSFET gate oxide and the resulting current mirror degradation by subjecting devices to TID doses from 50 krad(Si) to 300 krad(Si) using a 60Co gamma source Experimental data show that threshold voltage shifts by up to 1.31 V and transconductance increases by 27%. This degradation leads to this a reduction of more than 10% in current mirror output accuracy occurs at the highest dose. These quantitative criteria establish a clear benchmark for assessing the impact of TID on current mirror performance. These effects are attributed to positive charge trapping in the gate oxide and at the Si–SiO_2_ interface induced by ionizing radiation. This study focuses exclusively on radiation effects; electrical stress phenomena such as over−voltage or electrostatic discharge (ESD) are not addressed. The results highlight the critical importance of accounting for TID effects when designing high−performance n−MOSFET current mirrors for radiation−hardened applications.

## 1. Introduction

As semiconductor technology has advanced, integrated circuit (IC) applications have shown significant progress, while harsh environments, particularly space, have revealed critical challenges for device performance. Statistical analyses of satellite failures indicate that a major portion originates from electronic circuits rather than mechanical components [1,2]. Among these electronic circuits, MOS−based devices—including transistors, amplifiers, and current mirrors—play a pivotal role in analog and mixed−signal systems. Their proper functioning is crucial for tasks such as signal processing, biasing, and control in spaceborne electronics. Space radiation poses a significant threat to the reliable operation of electronic circuits, particularly MOS devices [3]. Exposure to radiation can induce Single−Event Effects (SEEs) and total ionizing dose (TID) effects, where TID refers to the cumulative ionizing radiation absorbed by the device over time, and SEEs correspond to instantaneous disruptions caused by energetic particles [3]. While much research has focused on SEEs, studies addressing TID mechanisms remain limited.

An inverter, as a fundamental building block of MOS circuits [4], is crucial for understanding overall system radiation tolerance. The TID effect is characterized by cumulative degradation of device performance due to high−energy photons and charged particles [5,6,7], primarily through positive charge accumulation in the oxide layer and interface trap formation at the SiO_2_/Si interface [8,9]. Unlike SEEs, TID cannot be completely mitigated due to its cumulative and physical nature. For electronic devices such as amplifiers, comparators, and voltage references, TID−induced shifts in threshold voltage or transconductance can propagate through circuits, causing timing errors, output drifts, and decreased amplification accuracy. Recent studies have modeled TID effects in advanced FDSOI technologies [10] and provided comprehensive analyses of radiation effects and soft errors in ICs [11].

Even at low doses, MOS devices are highly susceptible to radiation [12,13,14]. Beyond immediate performance impacts, long−term reliability post−radiation is critical, especially for space missions [15]. While TID is a key concern, intrinsic aging mechanisms such as Hot Carrier Injection (HCI), Bias Temperature Instability (BTI), and Time−Dependent Dielectric Breakdown (TDDB) [16,17,18,19] also affect device longevity independently. Though interface traps appear in these studies, their origins are typically process−related, thermal, or bias−induced, rather than radiation−induced. Nevertheless, TID and intrinsic mechanisms can produce similar effects, such as threshold voltage shifts and increased leakage, emphasizing the need for comprehensive reliability modeling.

Electrical stress, including BTI, can degrade MOS device parameters such as threshold voltage and transconductance [20,21,22,23,24]. When combined with TID, degradation may be exacerbated, leading to cascading effects in both analog and digital circuits. Accurate biasing is essential for proper IC operation [25,26,27]. Traditional CMOS dividers, based on resistors or transistors, have limitations due to process, voltage, and temperature dependencies [28]. Current mirrors (CMs) have emerged as efficient biasing elements capable of amplifying or transferring currents while providing better performance in terms of current gain, input−output impedance, and power consumption [29,30]. In electronic systems, current mirrors are frequently used to generate stable reference currents, drive active loads, and implement analog signal processing blocks, making them critical indicators of radiation−induced degradation. These characteristics make current mirrors essential for providing stable reference currents in analog circuits, which is particularly important when assessing TID−induced degradation in n−MOSFET devices.

This work focuses on investigating the TID−induced degradation of n−MOSFET−based current mirrors, with an emphasis on charge trapping mechanisms in the oxide layer under different biasing conditions. While electrical stress phenomena such as over−voltage or electrostatic discharge (ESD) are excluded, this study quantifies threshold voltage shifts and transconductance degradation under TID doses from 50 krad(Si) to 300 krad(Si) using a ^60Co gamma source, linking these effects directly to current mirror performance.

This paper is organized as follows: Section 1 provides a brief introduction and review of related works; Section 2 presents time−dependent hole trapping models and their impact on flat−band voltage shifts in the oxide layer; Section 3 describes reliability analyses of TID effects on n−MOSFET current mirrors; and Section 4 summarizes conclusions and perspectives.

## 2. Hole Trapping Models and Flat−Band Voltage Shift

### 2.1. Hole Trapping

Due to ionizing radiation, the charge build−up in the oxide layer arises from the generation of electron−hole pairs and subsequent hole trapping. This supposes that de−trapping of holes is neglected. Another simplifying hypothesis can also be adopted. It consists of assuming that the distribution of hole traps is uniform. This leads to the rate equations that govern the trapping of holes as follows:

(1) $$\frac{\partial p_{Trapped}(x,t)}{\partial t}=\frac{d\gamma }{dt}-v\frac{\partial p_{Free}(x,t)}{\partial x}$$

(2) $$\frac{\partial p_{Trapped}(x,t)}{\partial t}=\sigma vp_{Free}\left(x,t\right)[N_{T}-p_{Trapped}\left(x,t\right)]$$

where *γ* denotes the rate of generation of electron−hole pairs per unit volume, *v* is the velocity of holes, *σ* defines the capture cross−section of the trap sites, and *N_T_* is the density of hole traps throughout the oxide layer. Under this form, it is worth noting that Equations (1) and (2) can be solved exactly only when the term *γ* is not accounted for. An illustration is given in the report of Ning [30].Later, the charge build−up in MOS structures has been undertaken by Viswanathan [31].Interestingly, the rate equations were solved for γ non null, but in two limiting cases:

i—The density of trapped holes is not large compared to the total number of hole traps.

ii—The trapping efficiency is much lower than the rate of electron−hole generation. The solutions thus adopted in the last case can be combined under a general form:

(3) $$P_{Trappedholes}\left(x,t\right)=N\left(1-e^{-\frac{x}{\lambda }}\right),$$

with {*N = γ*; λ *=*$\;\frac{1}{N_{T}}$} in the first limiting case, and {*N =* $N_{T}$; λ  *=*$\;\frac{1}{\sigma \gamma }$} in the second one.

The origin of the *x*−axis is fixed at the gate−dioxide interface. It has been found that the density of trapped holes is exponentially high, with a saturated trend towards the silicon−dioxide interface. In Equation (3), λ can be interpreted as the probability per unit length of a hole being trapped within the oxide layer under irradiation.

### 2.2. Time−Dependent Hole Trap Density and Transconductance

Because of hole trapping, a potential shift can occur at the oxide terminal, and its expression versus trapped holes *p(x,t)* is given by ${∆V}_{FB}\left(t_{ox},\lambda \right)=-\frac{q}{\varepsilon_{0}\varepsilon_{rox}}\int_{0}^{t_{ox}}xP_{Trapped}\left(x,t\right)dx,$ where *q* is the electronic charge, *Ɛ_rox_* represents the dielectric constant of the oxide layer, Ɛ_0_ is the permittivity of free space, t_ox_ is the oxide thickness, and FB is a subscript representing a flat band. In evaluating the integral as a function of *t_o_*_x_, the mean trapping free length is treated as a parameter.

The calculation of ${∆F}_{FB}\left(t_{ox},\lambda \right)$ under a positive bias voltage leads to the following expression:

(4) $${∆V}_{FB}\left(t_{ox},\lambda \right)=-\frac{qNt_{ox}^{2}}{{Ɛ}_{0{Ɛ}_{rox}}}F(\frac{t_{ox}}{\lambda })$$

with

$$F\left(\frac{t_{ox}}{\lambda }\right)=\frac{1}{2}+\frac{\left(\frac{t_{ox}}{\lambda }+1\right)}{\frac{t_{ox}^{2}}{\lambda^{2}}}e^{-\frac{t_{ox}}{ʎ}}-\frac{1}{\frac{t_{ox}^{2}}{\lambda^{2}}}$$

For a fixed thickness of the oxide layer and taking the mean trapping free length *λ* to be less than *t_ox_*, the pre−exponential factor *N* will correspond to the hole trap density *NT*. On the other hand, by identifying Δ*V_FB_* for the threshold potential shift Δ*V_th_* (TID), as measured under irradiation, we can derive the TID−dependent oxide trapped charge density from the following relation:

(5) $$N_{T}\left(TID\right)\frac{{Ɛ}_{0}{Ɛ}_{rox}}{qt_{ox}^{2}F(\frac{t_{ox}}{\lambda })}{∆V}_{th}\left(TID\right)$$

To further improve the trapping charge model, it is required to assume the existence of an additional sheet charge near the *Si/SiO*_2_ interface, related to surface states or arising from shallow traps. The contribution of all these charge states can be approximately accounted for by defining the effective density of hole traps as follows: ***N**_**T**_^**EFF**^ = (***1** *+ **α**)**N**_**T**_*, where *α* represents a centesimal percent factor, which is negative if the additional sheet charge is predominated by electron−traps or positive in the reverse case. Here, this correction is not taken into account.

A great deal of interest is also paid to the transconductance parameter. Let *V_GS_ (t, TID) = V_GS_(t, TID =* 0*) + ∆V_FB_(t, TID)* be the gate−to−source bias voltage under gamma radiation. Similar to the hole trap density, the time−dependent transconductance is expressed as follows:

(6) $$g_{m}\left(TID\right)=\frac{g_{m}(TID=0)}{1-ɳ[\frac{qt_{ox}^{2}}{{Ɛ}_{0}{Ɛ}_{rox}}F(\frac{t_{ox}}{\lambda })]N_{T(TID)}}$$

where ɳ is an empirical factor, and *gm (TID = 0)* is the initial transconductance before irradiation. Notably, ɳ can be determined from the measured gm (TID) and the calculated *NT(TID)* using the following relation:

(7) $$ɳ=\frac{1-\frac{g_{m}(TID=0)}{g_{m}(TID)}}{\frac{qt_{ox}^{2}}{{Ɛ}_{0}{Ɛ}_{rox}}F(\frac{t_{ox}}{\lambda })N_{T(TID)}}$$

## 3. Reliability Analyses of Total Ionizing Dose Effects on n−MOSFET Current Mirrors

### 3.1. TID−Induced Electrical Degradation of an n−MOSFET

The n−MOSFET under investigation was fabricated using 1µm channel Silicon−On−Insulator (SOI) technology, with a gate oxide thickness of *t_ox_ =* 25 nm. Devices were prepared on a standard SOI substrate and underwent pre−irradiation conditioning to ensure uniformity. Details on the epitaxial growth and annealing process are provided in ref. [32]. This study focuses solely on TID effects, excluding electrical stress phenomena such as over−voltage or electrostatic discharge (ESD), to isolate the impact of TID−induced charge trapping on threshold voltage and transconductance. Long−term, time−dependent post−irradiation measurements were not conducted and will be addressed in future work to assess device reliability under prolonged TID exposure.

Concerning *VGS−IDS* characteristics, measurements were performed before and after irradiation. The n−MOSFET devices were subjected to gamma irradiation using a 60Co source. The total ionizing dose (TID) was incrementally increased up to 300 krad(Si). Measurements were performed at room temperature before and after each irradiation step, without any annealing before for VDS fixed at 50 mV in the linear regime and *VDS =* 3 V in the saturation regime. As it is seen, the threshold potential shows a negative shift with increased TID, and the relevant data are found to be fitted by the polynomial law:

𝚫𝐕𝐓𝐡= 𝟏.𝟔𝟗 – 𝟏𝟐𝟒𝟏𝟎 − 𝟓 × 𝐓𝐈𝐃(8)

where *V_th_* (*TID* = 0) = 1.69 V. From the measured *ΔVth(TID*), we deduced the density NT of induced hole traps as a function of TID using Equation (6). To highlight the impact of TID radiation, pre− and post−irradiation values of threshold voltage and transconductance were compared. The degradation of device performance is directly linked to hole trapping in the gate oxide. Ionizing radiation generates electron−hole pairs in the oxide, and the holes are captured at trap sites, resulting in a net positive charge. Simultaneously, interface traps form at the *Si–SiO*_2_ interface, further affecting the threshold voltage and transconductance. These trapped charges reduce effective gate control over the channel, leading to a measurable decrease in current mirror accuracy and overall device performance, as reflected in the observed Vth and g_m_ shifts.

The threshold voltage decreased from *V_th_ =* 1.69 V before irradiation to 1.32 V at the highest dose of 300 krad(Si), while transconductance increases from *g_m_* = 1.099 µS to 1.40 µS. These comparisons clearly illustrate the extent of TID−induced degradation in the n−MOSFETs and provide quantitative evidence for the charge trapping effects discussed above. The detailed results are summarized in Table 1**.**

**Figure 1:**
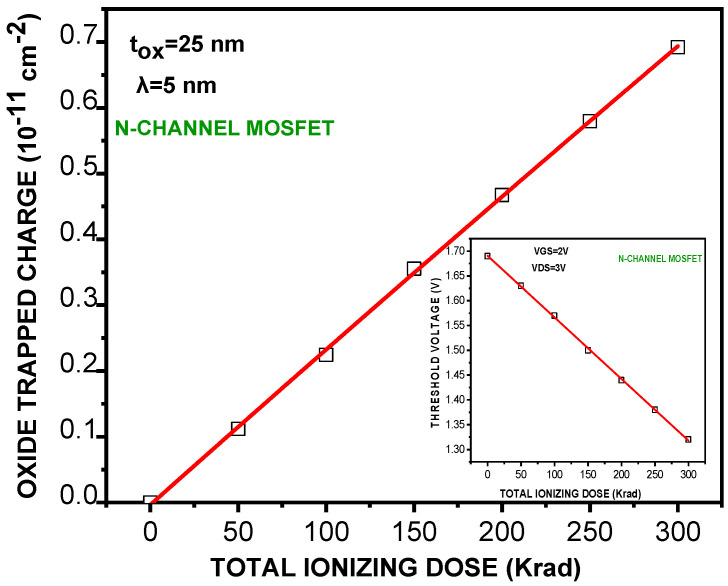
Evolution of oxide trapped charge density and threshold voltage value of an n-MOSFET (*L =* 1 µm, *W =* 24 µm, *t_ox_* = 25 nm) with increasing total ionizing dose (^60^Co γ source).

The mean trapping free length is fixed at **λ** = 5 nm, which corresponds to ${\;e}^{-\frac{t_{ox}}{\lambda }}=6\times 10^{-3}$. Results are depicted in Figure 1 for an interfacial charge plot ***N**_**T**_^**2D**^ = **N**_**T**_× **t**_**ox**_.* It is clear that there is a gradual increases in the amount of trapped holes as the total ionizing dose increases. As it has also been mentioned, the interfacial density of hole traps is related to TID by the following fitting relation:

𝐍_𝐓_^𝟐𝐃^(𝟏𝟎^𝟏𝟏^ 𝐜𝐦^−𝟐^) = −𝟑𝟓𝟔 × 𝟏𝟎^−𝟓^+ 𝟐𝟑𝟖 × 𝟏𝟎^−𝟓^× 𝐓𝐈𝐃 − 𝟏. 𝟕𝟖 × 𝟏𝟎^−𝟕^× 𝐓𝐈𝐃^𝟐^ in cm^−n^ (9)

**Figure 2:**
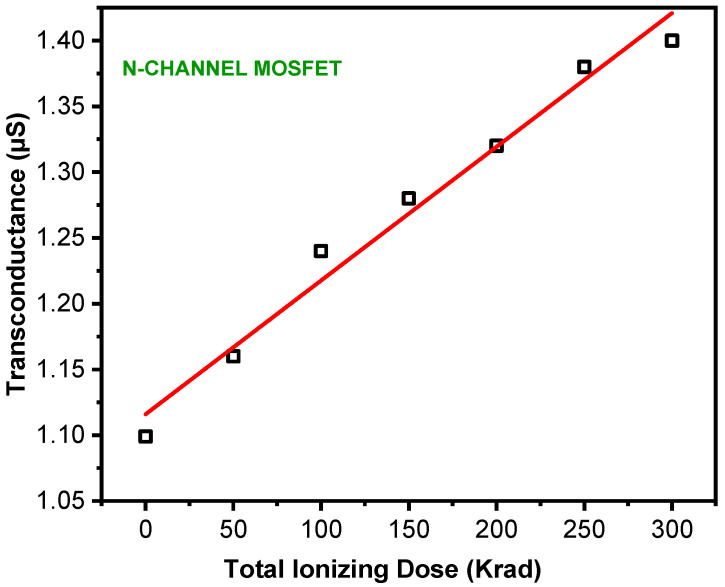
Transconductance (*g_m_*) as a function of TID for an n-MOSFET (L = 1 µm, W = 24 µm). Data were extracted at saturation regime with *V_DS_* = 3 V following different total dose levels.

In addition to *NT*, the graph shows the corresponding threshold potential versus TID. A proposed explanation is that exposure to relatively high TID levels induces defects in the MOS capacitance. This can make it difficult for electrons to flow into the conductive channel. Microscopically, an accumulation of holes reduces the switching speed of the MOSFET. As a peculiar feature, the threshold potential shift is found to be more significant at lower TID doses. Additionally, an increase in the threshold voltage can enhance the power consumption. Another fundamental parameter of the n−MOSFET that is impacted by gamma radiation is transconductance. Measurements of this parameter have led to an increasing trend versus TID. The data points are depicted in Figure 2 with the obtained fitting relation:

𝐠_𝐦_(𝐓𝐈𝐃) = 𝟏.𝟏𝟏 + 𝟏.𝟎𝟐𝟏𝟎^−𝟑^ × 𝐓𝐈𝐃 n **µS** (10)

From the measured *gm(TID*) and *NT(TID*), we have computed the empirical factor ɳ (TID). Results are found to be fitted using the following equation:

ɳ(𝟏𝟎^𝟏𝟓^ 𝐦^−𝟐^) = −𝟎.𝟎𝟒𝟐 − 𝟑.𝟑𝟓 × 𝟏𝟎^−𝟓^× 𝐓𝐈𝐃 + 𝟕 × 𝟏𝟎^−𝟕^ × 𝐓𝐈𝐃^𝟐^−𝟏.𝟒𝟓 × 𝟏𝟎^−𝟗^ × 𝐓𝐈𝐃^𝟑^ (11)

These observed degradations in threshold voltage *V_th_* and transconductance g_m_ can be directly linked to hole trapping mechanisms in the gate oxide and the formation of interface traps at the *Si–SiO*_2_ interface. As the total ionizing dose increases, the accumulation of positive charges in the oxide layer reduces the effective gate control over the channel, resulting in a negative shift in *V_th_* th. Simultaneously, trapped charges and interface states scatter carriers, decreasing the channel mobility and consequently the transconductance. This correlation between charge trapping and electrical parameter degradation provides a mechanistic explanation for the performance loss observed in n−MOSFETs under TID irradiation [30,31].

**Figure 3:**
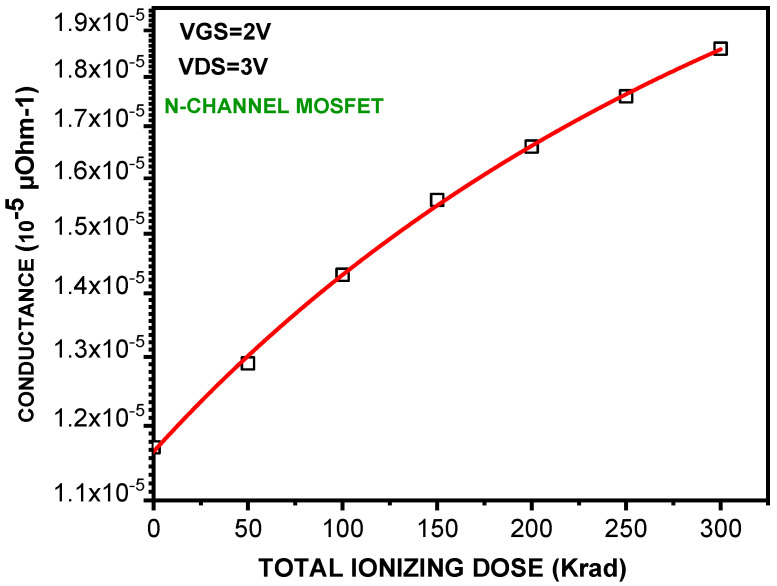
Drain conductance g_d_ versus TID for an n-MOSFET under increasing irradiation. Experimental points are shown with polynomial fitting (Equation (12)).

For the conductance, however, we did correlate this parameter with the charge build−up in the oxide. Calculations were performed using TID as an auxiliary variable, which has led to the plot of Figure 3. As it can be noticed, the conductance increases with TID according to the following fitting relation:

𝐠_𝐝_(𝐓𝐈𝐃) = 𝟏.𝟏𝟔𝟏𝟎^−𝟓^+ 𝟐.𝟖𝟑𝟏𝟎^−𝟖^ × 𝐓𝐈𝐃 − 𝟏𝟕𝟏𝟏𝟎^−𝟏𝟏^ × 𝐓𝐈𝐃 (12)

The conductance increases with TID, reflecting enhanced leakage paths induced by oxide charge trapping. Scientific notation is used for small conductance values to improve readability.

### 3.2. Electrical Degradation Mechanisms in n−MOSFET Current Mirrors

**Figure 4:**
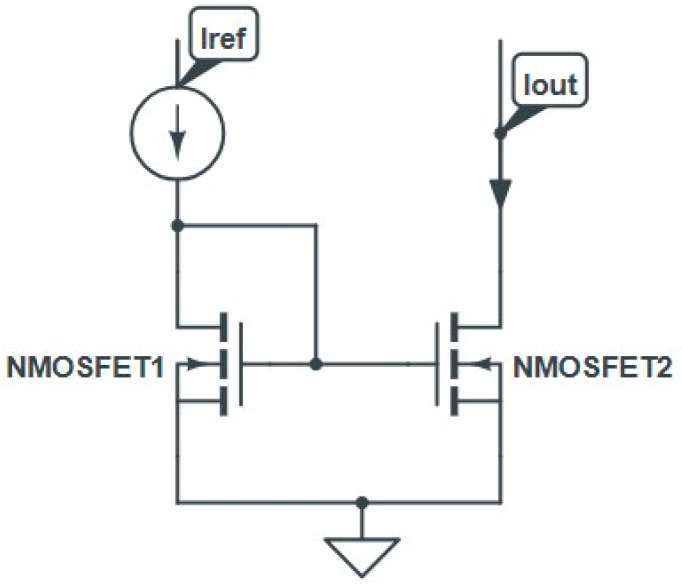
Schematic of a basic current mirror based on two nchannel CMOS transistors, NMOSFET1 (diode connected) and NMOSFET2.

The biasing of the CM as it used in AMS integrated circuits [29] is shown in Figure 4, with its dynamical equivalent scheme. It is constructed using two n−MOS transistors labeled M1 and M2.The diode−connected transistor M1 operates in saturation regime and converts the reference current *I_eEF_* into a corresponding gate−to−source voltage *V_GS1_*. The transistor M2 is a regenerating module that gives rise to an output current *I_OUT_*. If both the transistors M1 and M2 are matched, the gate−to−source biases *V_GS_*_1_ and *V_GS_*_2_ are equal, and then *I_eEF_* would be perfect at the output of CM. Under saturation conditions, the reference and output currents are given by the following set of electrical equations: $I_{REF=}{\frac{1}{2}{µ}_{n}C}_{ox}({\frac{w}{L})}_{1}{\left(V_{GS1}-V_{TH1}\right)}^{2}$ and $I_{OUT=}{\frac{1}{2}{µ}_{n}C}_{ox}({\frac{w}{L})}_{2}{\left(V_{GS2}-V_{TH2}\right)}^{2}$.

**Figure 5:**
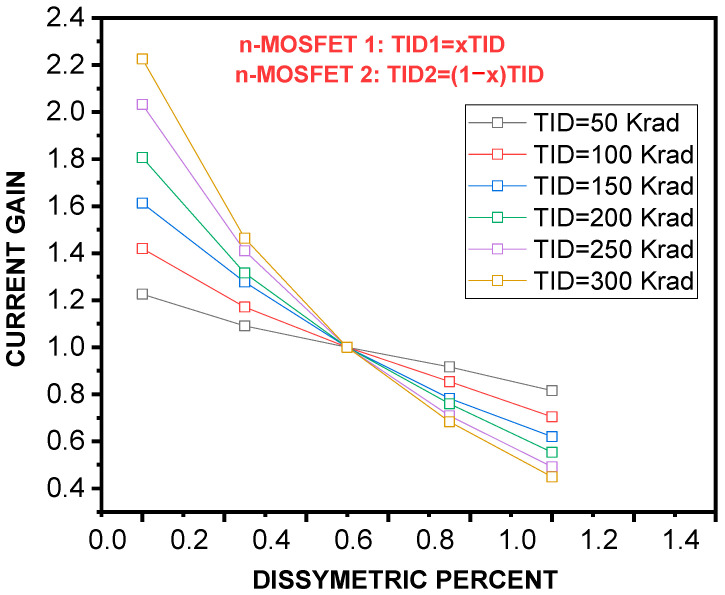
Current gain of a simple current mirror (SCM) as a function of dissymmetry percentage (x) at different TID levels. The gain decreases as TID increases, highlighting reduced current replication accuracy due to radiation−induced oxide charge trapping.

Where µ_n_ is the electron mobility, C_ox_ denotes the MOSFET capacitance per unit area, and *V_THi_ =* 1.2 is the threshold potential of the n−MOSFETs. From *I_REF_* and *I_out_*, we can derive the current gain. This parameter is undoubtedly affected by irradiation. Let *TID*1 *= x TID* and *TID*2 = (1 − *x*)*TID*, with 0 ≤ x ≤1 being the total ionizing doses for the MOSFET1 and MOSFET2 transistors, respectively. The current gain of the SCM reads is as follows: $A_{i(x,TID)}^{SCM}=\frac{({\frac{w}{L})}_{2}}{({\frac{w}{L})}_{1}}\times \frac{V_{GS}-V_{TH}[\left(1-x\right)TID\;]}{V_{GS}-V_{TH}[xTID]},$ where *x* represents a dissymmetric percent that characterizes the TID contamination of the n−MOSFETs. The calculation of *Ai^SCM^(x.TID)* has led to a graph of the current gain of an n−MOSCM versus x for different TIDs, as illustrated in Figure 5. We note that the current gain is a measure of how much an n−MOSFET mirror can replicate a current signal. The plot also shows that the current gain decreases as TID increases. This is because exposure to ionizing radiations damages the MOSFET’s gate oxide and reduces the ability of the gate to control the flow of current between the source and drain. As a further observation, the decreasing rate in current gain is found to change with the dissymmetric percent. Analytically, the x− and TID− dependent current gain are fitted according to the following equations:

(13) $$A_{i}^{SCM}\left(x,TID\right)=a_{0}+a_{1}\times TID+a_{2}\times {TID}^{2}$$

$$a_{0}\left(x\right)=1.017+0.00388\times x+2.1410^{-17}\times x^{2}$$

$$a_{1}\left(x\right)=-0.024-0.0088\times x+510^{-6}\times x^{2}$$

$$a_{2}\left(x\right)=-0.057+0.00229\times x+8.3510^{-6}\times x^{2}$$

Usually concerning the dissymmetric percent, it is worth noticing that a relatively high value of this coefficient means that the n−MOSFET gate−oxide becomes more asymmetric. This makes it sufficiently susceptible to submit damages from ionizing radiation. Two other important parameters garnered interest. They were input and output impedances of the SCM. According to the TD1 and TD2 defined above, both impedances are expressed as follows:

(14) $$R_{in}^{SCM}\left(x,TID\right)=\frac{1}{g_{m\left[xTID\right]}}$$

(15) $$R_{out}^{SCM}\left(x,TID\right)=\frac{1}{g_{d[(1-x)TID]}}$$

**Figure 6:**
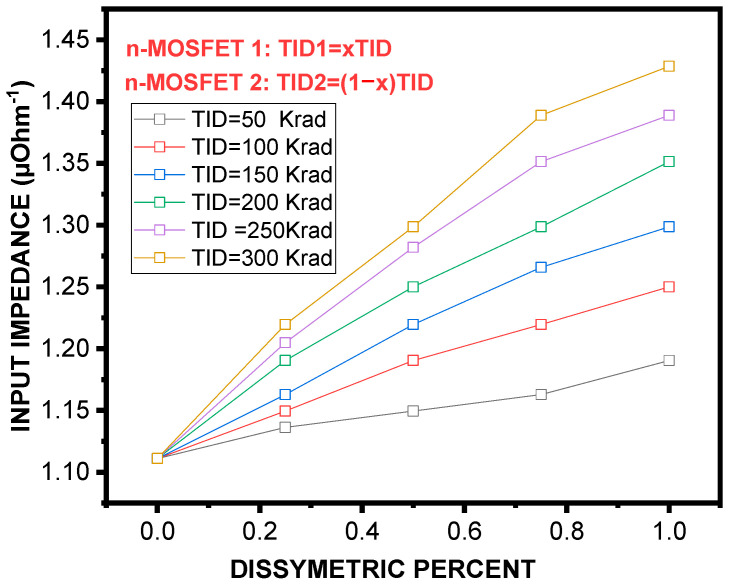
Input impedance of the SCM as a function of dissymmetry percentage under different TID doses. A positive correlation is observed, with both higher dissymmetry and larger TID contributing to increased input impedance.

**Figure 7:**
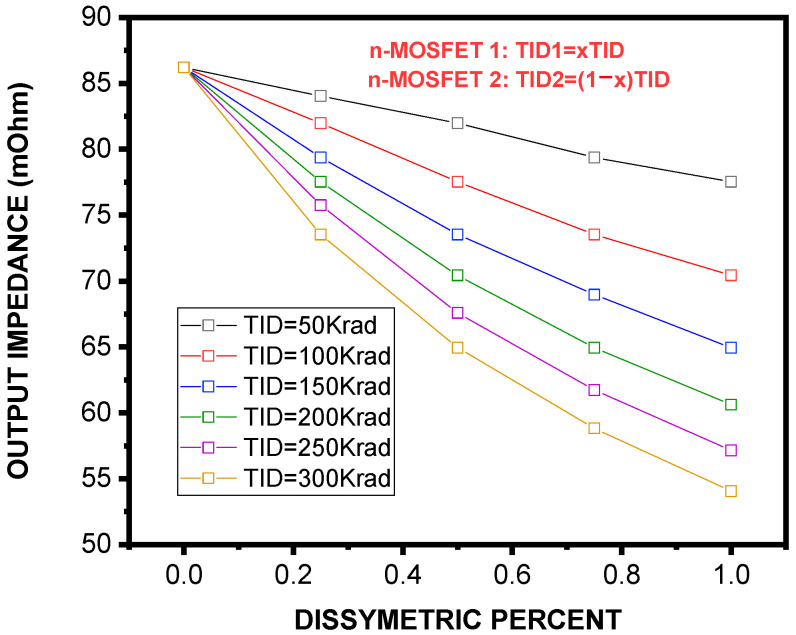
Output impedance of the SCM as a function of dissymmetry percentage under different TID doses. In contrast to the input impedance, the output impedance decreases with higher asymmetry and TID, reducing the ability of the current mirror to drive loads effectively.

Using Equations (14) and (15), we calculated *R^SCM^_in_* and *R^SCM^_ou_*_t_ versus x for different TIDs ranging from 50 Krad to 300 Krad. As it can be seen, Figure 6 shows a clear positive correlation between the dissymmetric percent and input impedance, independently of the TID level. Such an increase in impedance is assigned to the disruption of conduction paths produced by asymmetry. Moreover, the effect of TID is significant, suggesting that defects created by radiation also reinforce the CM resistance at input. The graph in Figure 7, however, shows how the output impedance is affected by the two factors. As it is seen, the impedance at output decreases with an increase in asymmetry percentage and total ionizing dose. The following set of expressions describes the analytical fitting of of ***R**^**SCM**^_**in**_(**x**, **TID**)* and ***R**^**SCM**^_**out**_(**x**, **TID**)*:

(16) $$R_{in}^{SCM}\left(x,TID\right)=a_{0}+a_{1}\times TID+a_{2}\times {TID}^{2}$$

$$a_{0}\left(x\right)=1.11-1.5510^{-18}\times x$$

$$a_{1}\left(x\right)=-7.9110^{-4}-0.00157\times x$$

$$a_{2}\left(x\right)=0.047-6.4510^{-4}\times x$$

(17) $$R_{out}^{SCM}\left(x,TID\right)=a_{0}+a_{1}\times TID+a_{2}\times {TID}^{2}$$

$$a_{0}\left(x\right)=86295.45-0.63\times x$$

$$a_{1}\left(x\right)=-1679.69-173.37\times x$$

$$a_{2}\left(x\right)=-4684.79+83.85\times x$$

In a concluding remark, the input and output impedances of a perfect current mirror should be very low and large enough, respectively. This suggests that degradations induced by geometric dissymmetry and the effect of ionizing radiation have a negative impact on CM’s ability to deliver more current to the load. It is then required to characterize these degradations in an attempt to develop an equivalent model that includes the parameter related to a radiation−hardened environment. In practice, SCMs are suitable for low−voltage applications. Technologically, the limited usage of SCMs contributed to their low output impedances. Some remedies were conceived to further improve the impedance at output. They consisted of Widlar CM [28] and cascade CM [27,28] designs. As it has been found, the output impedance of an SCM is increased due to Widlar and cascade arrangements based on the following ratios:

(18) $$\frac{R_{out}^{WMC}}{R_{out}^{SCM}}=1+Rg_{m2}\mathrm{and}\;\frac{R_{out}^{Cascode\;CM}}{R_{out}^{SCM}}={r_{ds3}g}_{m3}r_{DS2}g_{DS1}$$

In conclusion, it is worth mentioning that the cascade technology can open a promising way to construct electronic applications with low−voltage operation and low power consumption. But the use of complex configurations needs a multitude of elementary transistors. Because of different operating regimes, the cascade CMs could exhibit a mismatching in current and voltage between the input and the output. Exposure to ionizing radiations could also lead to further degradations.

### 3.3. Comparative Analysis with Literature

A comparison of our results with previous studies on TID effects in n−MOSFET devices is provided in Table 2. This table highlights differences in device types, TID dose ranges, measured parameters, and observed degradations, illustrating that n−MOSFET current mirrors experience substantial threshold voltage shifts and output accuracy loss compared to conventional MOSFETs reported in the literature.

## 4. Conclusions

In this study, we have investigated the combined effects of TID radiation and electrical stress on the performance of current mirrors based on n−MOSFETs. Through experimental measurements and simulations using LTspice, we inferred that both TID radiation and electrical stress independently contribute to the degradation of the current mirror’s accuracy, matching, and electrical performance. Our results demonstrate that TID radiation primarily causes charge trapping in the gate oxide, leading to a threshold voltage shift, while electrical stress provokes these effects by further altering device characteristics, such as transconductance and current gain. Consequently, current mirrors subjected to both TID radiation and electrical stress show significant changes in their characteristics, leading to a loss of precision and reliability. The findings highlight the importance of considering both radiation and electrical stress in the design and qualification of n−MOSFET current mirrors for radiation−hardened applications. To mitigate these effects, it is essential to explore design optimizations such as improved radiation−hardened materials, fault−tolerant circuit topologies, and enhanced device−shielding techniques. Further research should focus on developing a complete model to predict the combined impact of TID radiation and electrical stress. This undoubtedly allows to design more accurate MOSFET−circuits for high gamma−ray doses.

## Figures and Tables

**Table 1:** TID−induced degradation in n−MOSFET current mirrors.

TID Dose (krad(Si))	Threshold Voltage Vth (V)	Transconductance g_m_ (µS)
0	1.69	1.099
50	1.63	1.16
100	1.57	1.24
150	1.50	1.28
200	1.44	1.32
250	1.38	1.38
300	1.32	1.40

**Table 2:** Comparative summary of TID−induced threshold voltage and transconductance changes in n−MOSFETs and related devices.

Study/Reference	Device Type	TID Range (krad(Si))	Observed ΔV_th_ (V)	Observed g_m_ Change	Notes/Key Findings
This work	n−MOSFET current mirror, SOI, L = 1 µm, W = 24 µm	50–300	−0.37 (1.69 → 1.32)	+27% (1.099 → 1.40 µS)	Charge trapping in oxide, TID−induced degradation of current mirror accuracy
Cao et al., 2022 [2]	n−MOSFET	0–300	−0.3 approx.	+20%	Combined TID and electrical stress effects studied
Gao et al., 2023 [3]	CMOS inverter	0–150	−0.15	+10%	Simulation study of TID effects in inverter circuits
Bonaldo, 2022 [4]	GAA Si nanowire CMOS	0–500	−0.4	+25%	Ultra−high TID, highly scaled devices, significant ΔVth observed
Dubois et al., 2023 [7]	FDSOI 22 nm	0–200	−0.12	+8%	Modeling TID effects

## Data Availability

Data are contained within this article.
